# Natural infection of *Plasmodium falciparum* induces inhibitory antibodies to gametocyte development in human hosts

**DOI:** 10.1186/1475-2875-9-S2-P53

**Published:** 2010-10-20

**Authors:** Natda Tonwong, Jetsumon Sattabongkot, Jeeraphat Sirichaisinthop, Rachanee Udomsangpetch

**Affiliations:** 1 Department of Pathobiology, Mahidol University, Bangkok 10400, Thailand; 2 Department of Parasitology, Mahidol University, Bangkok 10700, Thailand; 3 Department of Entomology, Armed Forces Research Institute of Medical Sciences, Bangkok 10400, Thailand; 4 Vector Borne Disease Training Center, Pra Budhabat, Saraburi 18120, Thailand

## Background

**Figure 1:**
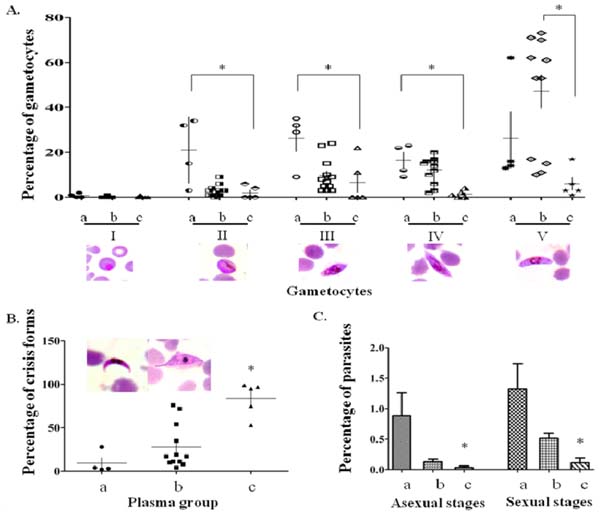
The effects of human plasma from malaria patients on maturation *of Plasmodium falciparum* gametocytes. The data are shown in (A) mean percentages of gametocytes from stage I to V, (B) mean percentages of crisis form of gametocytes, and (C) number of asexual and sexual stage parasites in the cocultivation cultures. (a) malaria naive plasma, (b) oocyst non-inhibitory plasma, (c) oocyst inhibitory plasma, *P ≤ 0.05.

Gametocyte antigens of *Plasmodium falciparum* can induce immunity in patients from which further inhibits fertilization of gametes, and consequently oocyst production in the mosquito midgut [1]. Here, we determined naturally-induced antibodies from malaria patients in Thailand and clarified effect of the antibodies on gametocyte development. Sixty-one percent of *P. falciparum*-infected blood fed to female Anopheles mosquitoes showed no oocyst production. Twenty-six percent of these oocyst inhibitory plasma distorted morphology and hampered maturity of the gametocytes (Fig.1). A possible mechanism of the gametocyte inhibitory activity was shown by binding of the plasma antibodies to the live immature intraerythrocytic gametocytes during co-cultivation period.

## Conclusion

Anti-gametocyte antibodies were elicited during natural malaria infection. The oocyst inhibitory antibodies diffused to and interacted with developing intraerythrocytic gametocytes and reduced number of stage II to V gametocytes, and hampered their maturation. Therefore the alternative development of transmission blocking vaccine in the high transmission area should focus on the identification of the gametocyte antigens inducing inhibitory antibodies to reduce gametocytemia.
